# Clinical characteristics for conservative therapy of pediatric parapharyngeal abscesses

**DOI:** 10.1016/j.bjorl.2019.10.015

**Published:** 2019-12-23

**Authors:** Jing Bi, Xiaowei Chen, Zhiying Zhou, Yong Fu

**Affiliations:** Children's Hospital of Zhejiang University School of Medicine, Department of Otolaryngology Head and Neck Surgery, Hangzhou, China

**Keywords:** Deep neck infection, Parapharyngeal abscess, Pediatric, Therapy

## Abstract

**Introduction:**

The role of surgical drainage versus conservative therapy in treating patients with parapharyngeal abscesses is still a theme of debate.

**Objectives:**

This study aimed to investigate the characteristics associated with good outcomes in pediatric patients with parapharyngeal abscesses treated with conservative therapy.

**Methods:**

This retrospective chart review was performed on children aged 0.3–14 years with the diagnosis of parapharyngeal abscesses confirmed by computed tomography from January 2013 to March 2018. Patients with a severe upper airway obstruction required early intervention, while those in a stable condition initially received conservative therapy with antibiotics. If the patients appeared unlikely to recover, additional surgical drainage was provided. Multivariate logistic regression models were constructed to investigate the clinical characteristics associated with a good response to conservative therapy. A receiver operating characteristic curve was used to identify the age and abscess size cutoff for predicting a successful response.

**Results:**

A total of 48 children were included in the study. Patient age, antecedent illness, and abscess size were significantly associated with a response to therapy (Odds Ratio = 1.326, 2.314 and 1.235, respectively). The age cutoff associated with the conservative therapy was 4.2 years (76.9% sensitivity, 68.2% specificity), and the abscess size cutoff associated with the conservative therapy was 23 mm (84.6% sensitivity, 77.3% specificity).

**Conclusion:**

The findings suggested that younger age, smaller abscess size, and less frequent antecedent illnesses, such as upper respiratory tract infection and lymphadenitis, could predict a successful response to conservative therapy in pediatric patients with parapharyngeal abscesses.

## Introduction

Parapharyngeal abscess (PPA) is a common but dangerous deep neck infection in pediatric patients. It can result in local, regional, and systemic complications, including airway obstruction, mediastinitis, and paraspinal abscesses, leading to paralysis, jugular vein thrombophlebitis, cranial nerve dysfunction, cervical osteomyelitis, meningitis, and death.^1^ The incidence of PPA has increased in children in recent decades.^2^, ^3^ In younger children, the most common presentations are fever and reduced appetite rather than a sore throat, while airway obstruction, neck swelling, and tenderness are also common presentations of PPA. Early diagnosis and treatment are critical to prevent such conditions and improve patient survival. Because of lower compliance and smaller anatomy, a pediatric PPA demands special skills in terms of diagnosis and treatment.

The conventional treatment for PPA in adults is antibiotic therapy with surgical drainage, whereas in children, the role of surgical drainage versus conservative therapy is still debatable.^4^ Some studies suggested that antibiotic therapy could be used alone if no evidence of a compromised airway is found.^5^, ^6^, ^7^, ^8^, ^9^ Danny et al.^7^ reported that deep neck abscesses (including retropharyngeal and parapharyngeal abscesses) with smaller abscesses (≤25 mm maximal diameter) and younger age respond to antibiotics as the first-line treatment. Dong-Kyu et al.^10^ found that younger age, fewer episodes of acute tonsillitis, and smaller abscess size could predict the favorable outcome of nonsurgical treatment of a pediatric peritonsillar abscess. To date, the indications for conservative therapy of PPA in pediatric patients remain unknown. Therefore, this study was performed to investigate the characteristics associated with good outcomes in pediatric patients with parapharyngeal abscesses treated with conservative therapy by analyzing 5 year data from the Zhejiang University.

## Methods

### Patients

This retrospective study included consecutive patients who visited either the clinical department for the therapy of parapharyngeal abscesses or the emergency department for the therapy of severe upper airway obstruction from January 2013 to March 2018. Children aged 0.3–14 years were included. The diagnosis was confirmed via a Computed Tomography (CT) scan. Abscess size referred to the presence of frank pus, not just the murky fluid. Patients with obvious symptoms of upper airway obstruction received surgical drainage directly using intraoral and lateral neck approaches. Patients with no symptoms or signs of upper airway obstruction and systemic toxicity were admitted for close observation and intravenous antibiotic treatment. All patients underwent ultrasound-guided puncture of pus (ultrasound shows the hypoechoic liquid dark area of the parapharyngeal space, with no blood flow signal, but the wall of the abscess may have a low blood flow signal due to inflammation), bacterial culture, and drug sensitivity test based on the results of the selection of antibiotics. If the patients appeared unlikely to recover and had a persistent high fever, systemic toxicity, increased neck swelling, frank pus on a CT scan, or signs of upper airway obstruction after 24 h of intensive medical therapy, open surgical treatments such as drainage were provided. The exclusion criteria were as follows: (1) aged 15 years or older and (2) had a previous diagnosis of parapharyngeal abscesses. This retrospective study was approved by the Ethics Committee of local hospital.

### Data collection

All clinical information was obtained through a retrospective medical chart review, including age, gender, body mass index (BMI), presenting symptoms, physical examination, laboratory studies, radiological findings, antecedent illnesses, management, microbiology, and hospital duration. The abscess size was assessed based on radiological examination and C-Reactive Protein (CRP) levels. Also, bacterial culture results were obtained from swabs taken intraoperatively.

### Statistical analysis

A total of 48 children were divided into conservative therapy and surgical drainage groups. The Mann–Whitney *U* test or the Student *t* test was used to compare differences in clinical parameters between the two groups. The Pearson’s chi-square test was also used to evaluate associations. Additionally, multivariate logistic regression models were constructed to identify the predictors of a good response to medical therapy after adjusting for confounding factors. A Receiver Operating Characteristic (ROC) curve was used to determine the cutoff point of age and abscess size associated with a good response to conservative treatment. SPSS statistical software version 20.0 (SPSS Inc., IBM, USA) was used to conduct statistical analyses. A *P* value <0.05 was considered statistically significant.

## Results

A total of 48 children (30 male and 18 female) diagnosed with parapharyngeal abscesses were enrolled in this study. Their mean age was 4.5 years (range: 0.3–14 years). The clinical characteristics of the two groups are presented in Table 1.

**Table 1:** Clinical characteristic of pediatric patients with parapharyngeal abscesses.

Category	Conservative therapy	Surgical drainage	*p*-value
n	22	26	
Age, mean (SD)	3.7 (SD 2.4)	5.1 (SD 4.8)	0.002
Gender (male), n (%)	13 (59.1)	17 (65.4)	0.413
BMI, kg/m^2^	16.5 ± 2.4	17.6 ± 2.9	0.167
Abscess size, mm^2^	7.4 ± 2.1	12.1 ± 4.9	0.004
Hospital duration, d	10.6 ± 4.2	9.7 ± 4.5	0.134
C-reactive protein	75.8 ± 53.8	72.6 ± 58.4	0.643
Antecedent illness, n (%)			
Recent URI	5 (22.7)	4 (15.4)	0.652
Lymphadenitis	13 (54.5)	8 (34.6)	0.131
PSF	2 (4.2)	5 (8.3)	0.014
Leukemia	1 (4.5)	4 (15.4)	0.011
KD	1 (4.5)	5 (19.2)	0.023

BMI, Body Mass Index; KD, Kawasaki Disease; PSF, Pyriform Sinus Fistula; SD, Standard Deviation; URI, Upper Respiratory tract Infection.

No significant differences were found in gender (*p* = 0.413) and BMI (*p* = 0.167) between the two groups. Also, no significant differences in serum CRP values (*p* = 0.643) and hospital duration (*p* = 0.643) were observed between conservative and surgical treatments. Meanwhile, antecedent illnesses [leukemia, Kawasaki Disease (KD)] (*p* ＜ 0.05) and abscess size (*p* = 0.004) showed significant differences between the two groups.

Logistic regression models demonstrated that age, antecedent illnesses (leukemia and KD), and abscess size were significantly associated with a response to intensive medical therapy. The strongest association was found for antecedent illnesses (Odds Ratio ‒ OR = 2.314, 95% Confidence Interval ‒ 95% CI: 1.239–3.437) (Table 2).

**Table 2:** Multivariate logistic regression analyses to identify factors associated with conservative treatment in children with parapharyngeal abscesses.

Variables	OR	95% CI	*p*-value
Age	1.326	1.204–1.613	0.003
Gender	1.096	0.784–6.744	0.598
BMI	0.878	0.723–1.131	0.127
Abscess size	1.235	1.097–1.672	0.006
Antecedent illness	2.314	1.239–3.437	0.005
C-reactive protein	0.898	0.976–1.102	0.078
Hospital duration	1.936	0.975–3.496	0.087

BMI, Body Mass Index; CI, Confidence Interval; OR, Odds Ratio.

**Figure 1:**
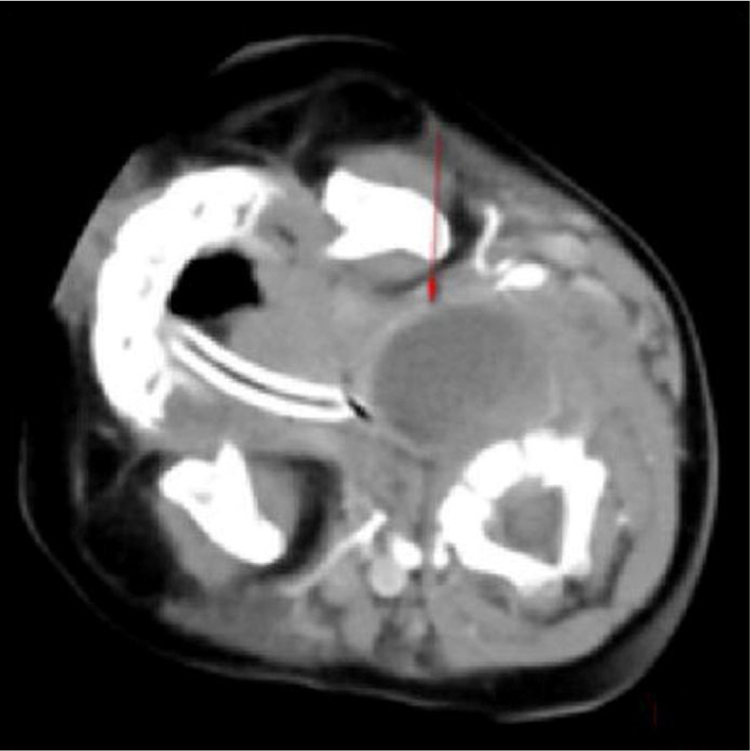
Left parapharyngeal abscesses with clear respiratory obstruction symptoms.

**Figure 2:**
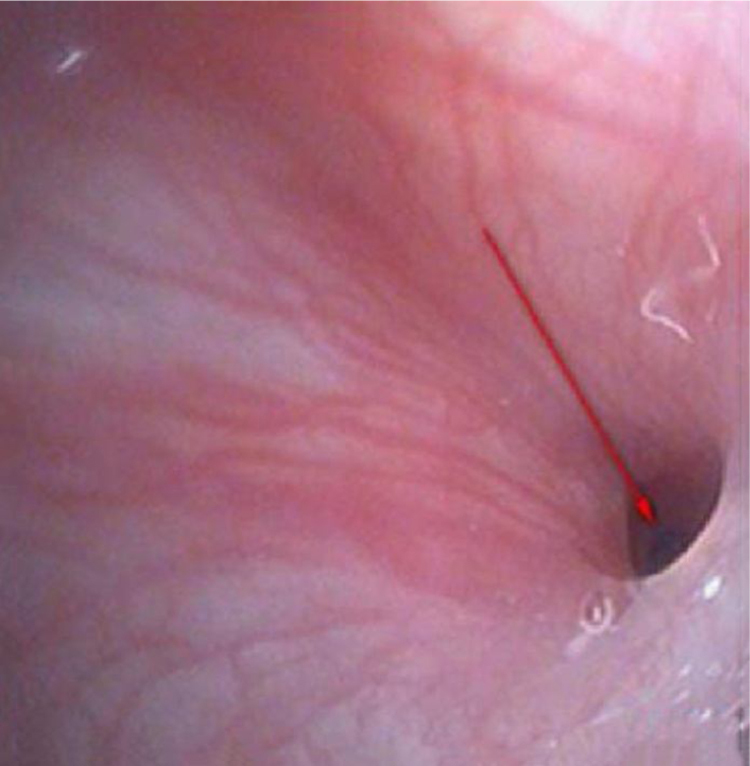
Laryngoscopic examination.

**Figure 3:**
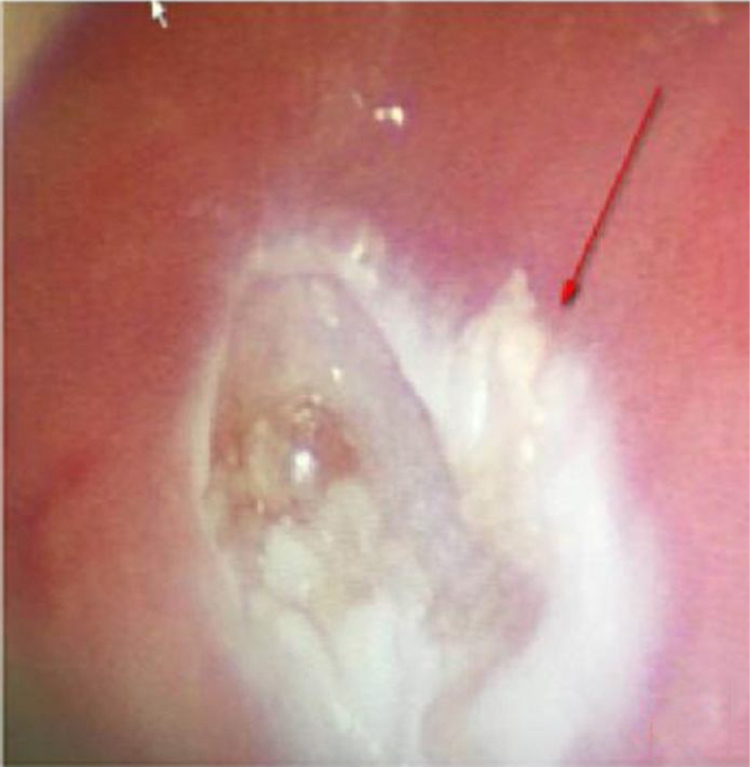
After burning the mouth of the inner fistula.

**Figure 4:**
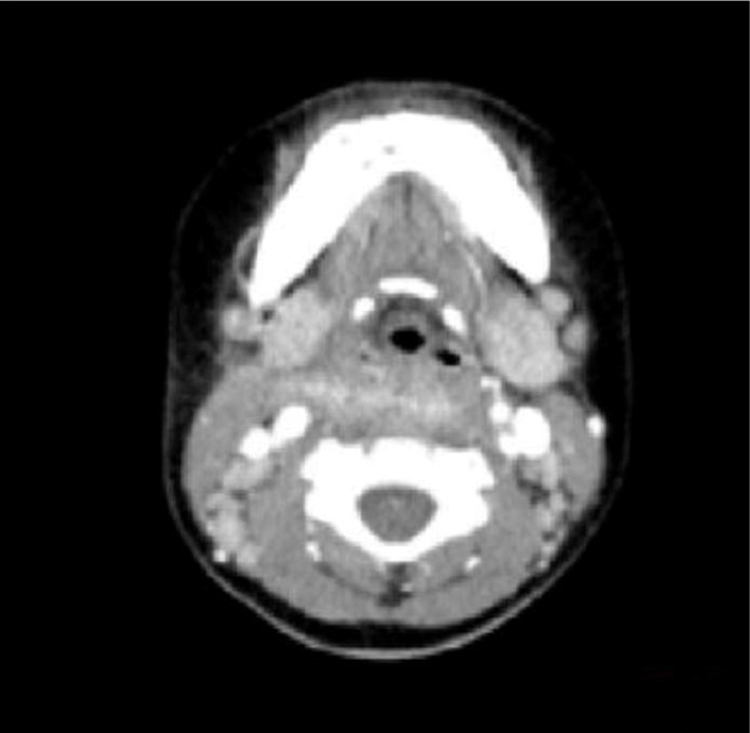
Review of the neck CT scan, the neck soft tissue is slightly swollen.

**Figure 5:**
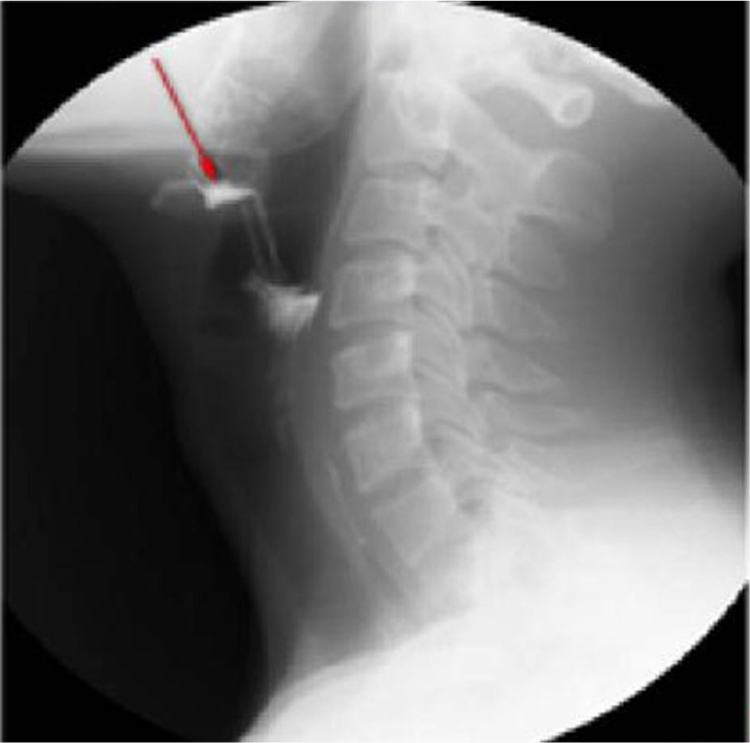
Esophagus imaging: a fistula from the left piriform fossa to the superior thyroid gland.

Of 36 children, 7 had Pyriform Sinus Fistula (PSF), and 5 underwent drainage. Clear respiratory obstruction symptoms were evident during the first visit. The patients were provided smooth breathing through tracheal intubation. However, the 24 h effect of conservative treatment was not good. The laryngoscopic examination confirmed the diagnosis of left PSF, which improved after cutting the drain and cauterizing the mouth of the inner fistula (Figs. 1‒4 ). Two cases improved with conservative treatment, followed by discharge. Esophagus imaging revealed left PSF (Fig. 5).

**Figure 6:**
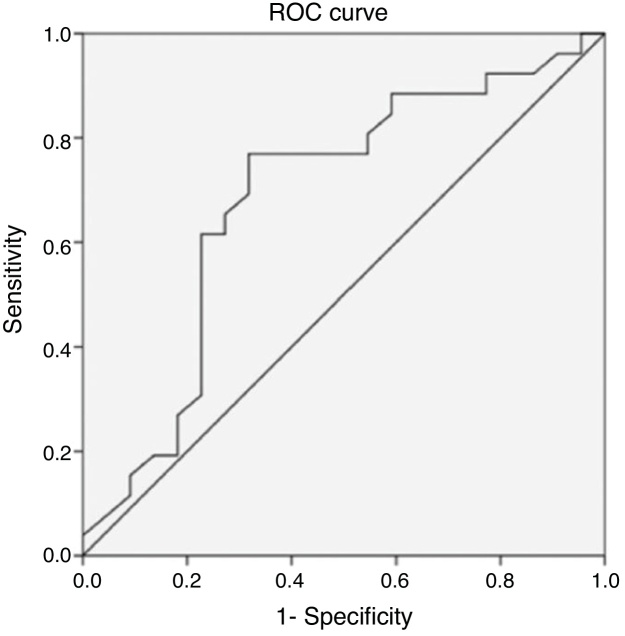
Receiver operating characteristic curve of the child’s age according to conservative treatment in pediatric parapharyngeal abscesses.

**Figure 7:**
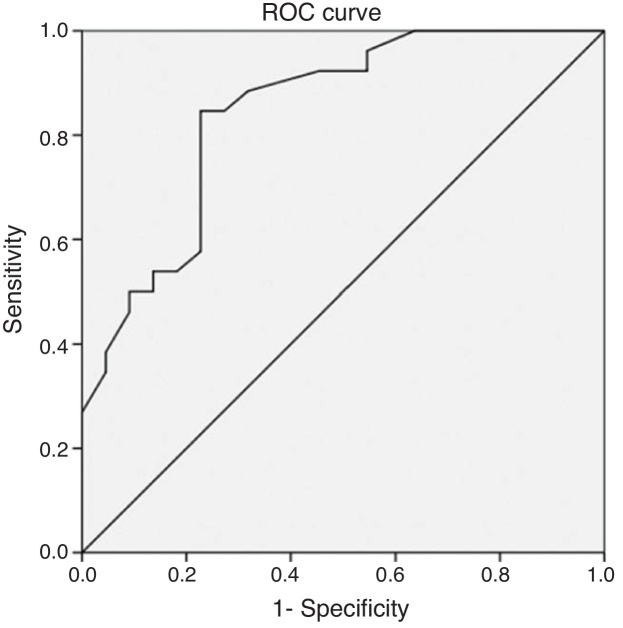
Receiver operating characteristic curve of the abscess size according to conservative treatment in pediatric parapharyngeal abscesses.

The ROC curve analysis was used to evaluate the characteristics of the predictors of a good response to intensive medical therapy. The ROC curve analysis demonstrated the cutoff age for surgery drainage as 4.2 years (76.9% sensitivity, 68.2% specificity). The area under the ROC curve for conservation was 0.684 (95% CI: 0.526–0.843, *p* = 0.029, *p* < 0.005) (Fig. 6). The ROC curve analysis demonstrated the cutoff abscess size for surgery drainage as 23 mm (84.6% sensitivity, 77.3% specificity). The area under the ROC curve for conservation was 0.844 (95% CI: 0.732–0.955, *p* < 0.005) (Fig. 7).

In addition, bacterial culture results were obtained from swabs taken intraoperatively, which were positive in 11 patients (42.3%). Of these, most patients showed the growth of *Streptococcus pyogenes* or *Staphylococcus aureus*, and one patient had methicillin-resistant *S. aureus*, they received therapy in the intensive care unit (Table 3).

**Table 3:** Microbiology of pediatric parapharyngeal abscesses (of 26 patients undergoing surgery treatment).

Microorganisms	Nº of cases
*Streptococcus pyogenes*	5
*Staphylococcus aureus*	3
Anaerobic bacteria	2
MRSA	1
No growth	12
No microbiology results	3

MRSA, Methicillin-Resistant *S. Aureus.*

## Discussion

The incidence of parapharyngeal abscesses in children is approximately 17–35 cases per 100,000 children per year,^11^ and it has increased recently.^2^, ^3^ The diagnosis and treatment are different from those in adults, and the infection can more easily spread into other deep neck spaces and result in life-threatening complications in children. Because of the patient’s low compliance and smaller anatomy, a pediatric PPA involves more special skills and challenges for pediatricians. To date, a large number of studies have been performed on pediatric deep neck infection. However, studies on pediatric PPA are rare, and most of these have presented only descriptive data, including epidemiology, age, sex, symptoms, seasons, microbiology, and admission course. In contrast, the present study focused on treatment decisions (medical versus surgical) in the pediatric population to allow for the recommendation of the appropriate treatment for PPA. This was the first large-population study concerning treatment decisions on pediatric PPA. The study showed that the clinical factor for a good response to successful conservative treatment was significantly associated with age, abscess size, and antecedent illnesses (leukemia and KD).

A previous study reported that children aged 6 years better responded to medical treatment compared with older groups.^1^ Another study found that the clinical presentation of pediatric PPA differed between children aged 5 years and <5 years.^12^ The ROC analysis also indicated that the cutoff age for a poor response to conservative treatment in children with PPA was 4.2 years (76.9% sensitivity, 68.2% specificity). The area under the ROC curve for conservation was 0.684 (95% CI: 0.526–0.843, *p* = 0.029, *p* < 0.005). Therefore, the findings suggested that pediatricians should consider nonsurgical treatment for younger children. The reason is unclear and may be related to age sensitivity to antibiotics.

Some studies revealed that the most frequent antecedent illness in pediatric PPA was lymphadenitis, whereas acute pharyngitis, including peritonsillar abscess, was the most frequent in adults.^13^, ^14^ The present study results (including 21 patients with lymphadenitis, 43.75%) were consistent with the findings of the aforementioned studies. Younger children with leukemia are more likely to develop infections in the parapharyngeal space and lateral neck because of less resistance, suppurative change, or lymphogenous spread of infections.^15^, ^16^ In addition, the present study showed that most patients were unresponsive to empiric antibiotics and needed surgery (*p* = 0.011, *p* < 0.05). Kawasaki disease, or mucocutaneous lymph node syndrome, is an acute multisystem vasculitis of unknown etiology that typically affects young children. Several reports are available on patients with KD having deep neck infections, such as peritonsillar abscess, suppurative parapharyngeal infection, or retropharyngeal abscess.^17^, ^18^, ^19^, ^20^ The mechanism via which KD causes parapharyngeal infection is unclear. It is speculated the inflammation causes increased vascular permeability, leading to protein exudation and ultimately local edema. The pharyngeal gap is a loose connective tissue more prone to edema. Swollen lymph nodes caused by lymphatic circulation disorders and lymphatic fluid accumulation in the pharyngeal gap may lead to a low-density edema region and abscess formation. The results of the present study are consistent with the aforementioned reports. Therefore, children diagnosed with KD having parapharyngeal abscesses need surgical treatment (*p* < 0.05). Pyriform Sinus Fistula (PSF) is a congenital neck-induced malformation caused by the insufficiency of the third and fourth incomplete rupture of the fissure in the early stages of embryonic development. The fistula formed by abnormal embryonic development is closely related to the adjacent structure of the cricoarytenoid joint area, forming the anatomical basis of secondary neck infection. PSF can cause repeated inflammatory lumps in the neck, acute suppurative thyroiditis or deep abscess of the lower neck.^21^ The clinical incidence of the disease is low due to the lack of the understanding of the disease, and it is more easily misdiagnosed. In the present group of seven children, five received surgical treatment, drainage, and endoscopic examination for clear diagnosis at the same time (Fig. 2). The patients showed improvement later (Figure 3, Figure 4). Two who received conservative treatment were discharged from the hospital after the follow-up and visited the department 1 month later for review. Esophagus imaging suggested left PSF (Fig. 5); the current follow-up did not recur. This suggested that the existence of PSF should be considered in the clinical work for pediatric patients with left PPA, and drainage should be performed while supporting the larynx to detect the mouth of the inner fistula to avoid misdiagnosis.

Some reports of successful conservative management have concentrated on abscess size, which has been shown to be an important predictor of treatment modality or the need for surgical intervention. Danny et al.^7^ reported deep neck abscesses (including retropharyngeal and parapharyngeal abscesses) with smaller abscesses (≤25 mm maximal diameter) and younger age responded to antibiotics as the first-line treatment. Hoffmann et al.^22^ reported that a long axis of 20 mm was more common for children to receive surgery after a period of medical management. However, high-quality evidence suggesting any particular characteristics only for PPA in children is lacking. In the present study, the ROC analysis indicated that patients were statistically more likely to have successful conservative management when abscesses were <23 mm (84.6% sensitivity, 77.3% specificity).

The present study reported the presence of aerobic organisms predominantly in pediatric PPA. Particularly *Staphylococcus* species (30.8%), anaerobic bacteria, and MRSA were uncommon, which was consistent with previous findings.^1^, ^6^, ^7^ Regarding antibiotic treatment, the patients were treated with penicillins and β-lactamase inhibitors (e.g., ampicillin/sulbactam) for the aforementioned organisms as soon as the diagnosis was made. Therapy might be altered later according to microbiological examinations and antibiograms if necessary. The incidence of community-acquired MRSA infection is rare, and hence anti-MRSA drugs were not used empirically.

The present study had several limitations. First, it was a retrospective study with a small sample size, short observation time, and findings from a single medical center. Second, the duration of hospital therapy in this study was insufficient to improve a patient’s condition. However, it was difficult to wait for more than 72 h because a local infection might suddenly become a systemic infection, and some pediatric patients might suffer from life-threatening airway obstruction. Third, empirical antimicrobial coverage might have affected the microbiological findings. Further studies should involve multicenter research, prolonged observation, and more information on factors such as socioeconomic status and the exact time of symptom onset.

## Conclusions

This study found that younger age, smaller abscesses, and fewer antecedent illnesses were significant predictors for choosing conservative treatment in pediatric parapharyngeal abscesses. It also suggested that the favorable response of nonsurgical treatment significantly diminished after 4.2 years of age and for abscess size larger than 23 mm. Therefore, intravenous antibiotics with analgesia and careful monitoring for airway or neurological complications may be considered as the initial treatment for young children presenting with a small abscess.

## Funding

This work was supported by the Healthy Department Project of Zhejiang Province (2018KY452).The authors have no other funding, financial relationships, or conflicts of interest to disclose.

## Conflict of interest

The authors declare no conflict of interest.
